# Contraception, female cycle disorders and injuries in Swiss female elite athletes—a cross sectional study

**DOI:** 10.3389/fphys.2023.1232656

**Published:** 2023-07-26

**Authors:** Sabrina Baumgartner, Norman Bitterlich, Sarah Geboltsberger, Maja Neuenschwander, Sibylle Matter, Petra Stute

**Affiliations:** ^1^ Department of Obstetrics and Gynecology, Inselspital, University of Bern, Bern, Switzerland; ^2^ Swiss Federal Institute of Sport—BASPO, Magglingen, Switzerland; ^3^ Retired, Chemnitz, Germany; ^4^ Swiss Olympic, Ittigen, Switzerland; ^5^ Medbase Sports Medical Center, Bern, Switzerland

**Keywords:** female cycle disorder, contraception, lean sports, injuries, amenorrhoea

## Abstract

**Aim:** The national Olympic committee of Switzerland has conducted an online survey among female elite athletes with a focus on cycle disorders, contraception, and injuries in 2021.

**Methods:** A total of 1,092 female elite athletes from 107 different sports were asked to answer the questionnaire. A descriptive analysis was carried out to determine location parameters and create frequency tables.

**Results:** The questionnaire was completed by 408 athletes (37.4%) from 92 different sports. 43.4% participated in a lean sport. 57.1% reported no injuries, 32.6% one injury, and 10.2% two or more injuries per year. A considerable proportion reported being affected by primary amenorrhoea (10.8%). Primary amenorrhoea occurred significantly more often in female athletes with a BMI lower than 21.7 kg/m^2^ (15.2%) than in athletes with a BMI above 21.7 kg/m^2^ (7.4%, *p* = 0.021). Considering contraception, 25.8% of female athletes were currently using an oral contraceptive pill. The proportion of female athletes not using contraception at all or using non-hormonal contraceptive methods was high at 54.4%. In lean sports, significantly more athletes used no or non-hormonal contraceptives (*p* < 0.05).

**Conclusion:** Among top Swiss female athletes, a considerable proportion used non-hormonal or no contraceptives. This trend was more evident in lean sports. Delayed menarche and cycle irregularities were common among female athletes, especially among athletes with high training volumes as well as a BMI below 21.7 kg/m^2^. This orienting survey underlines the importance of specialized gynecological care for elite female athletes.

## 1 Introduction

Optimizing performance is the focus of every athlete, who will usually aim to improve it through a balanced cycle of training and recovery phases. In so-called lean sports, performance is linked to body weight and body composition. This is reflected in a higher rate of cycle disorders (up to 79%) in these sports (Torstveit and Sundgot-Borgen, 2005a). Lean sports emphasise achieving and maintaining the ideal body type or body weight for the sport, to improve performance. Lean sports can be further divided into aesthetic sports, endurance sports, sports with required weight categories, and antigravity sports. In contrast, power sports, ball sports, and technical sports are the main representatives of “non-lean sports” (Mancine et al., 2020). The pursuit of an ideal body and an improvement in physical performance, often combined with perfectionist personality traits, is associated with an increased risk of these athletes developing a conscious or unconscious energy deficiency or even an eating disorder (Werner et al., 2013). An energy deficiency in turn triggers a metabolic cascade that disrupts the hypothalamic-pituitary-gonadal (HPG) axis, which can result in hypothalamic hypogonadism and cycle disorders, extending through to complete amenorrhoea. In addition to the inhibited HPG axis, an energy deficiency also leads to a dysfunction of other body systems. These include reduced metabolic rate, impaired bone health, compromised immune system, psychological problems, reduced protein synthesis, and increased cardiovascular risk (Mountjoy et al., 2018). The condition of reduced bone density and absence of menstruation in the context of reduced caloric intake or an eating disorder in female athletes was previously referred to as the Female Athlete Triad (FAT) by The American College of Sports Medicine (Nattiv et al., 2007). The International Olympic Committee) consensus group has revised this concept and replaced it with the term RED-S (Relative Energy Deficiency in Sports), a broader term that encompasses all associated conditions and includes male athletes (Mountjoy et al., 2014). The risk of low energy availability with consequent RED-S or FAT is higher in individual sports and lean sports (Mancine et al., 2020). Depending on the type of sport, up to 61% of the female athletes surveyed were affected by amenorrhoea (primary amenorrhoea, secondary amenorrhoea) (Redman and Loucks, 2005; Gibbs et al., 2013), compared to 3%–4% in the normal population (Pettersson et al., 1973; Bachmann and Kemmann, 1982; Nawaz and Rogol, 2022). The latter statistic comes from a major survey conducted 20 years ago on cycle disorders and the risk of energy deficiency among top Swiss female athletes (Matter and Marti, 2001). The present survey aimed to analyse issues in the field of “women`s health and elite sports” and to provide up-to-date data on the following key topics: 1) cycle disorders, 2) contraception, and 3) injuries which were further correlated with the type of sport (lean *versus* non-lean, team *versus* individual) and subjective characteristics.

## 2 Materials and methods

### 2.1 Sample and procedure

In spring 2021, 1,092 female elite athletes from 107 different sports received an invitation from Swiss Olympic to participate in the study. The invitation was part of a periodic newsletter that was sent by email to the female elite athletes. In the email newsletter, the athletes were invited to participate in the study. The attached documents took the athletes directly to the study information including a direct link to participate in the anonymous electronic survey. The selected athletes were competing at least at an international level. According to the responsible Ethics Committee, no ethical approval was required to conduct this survey. The classification into lean *versus* non-lean sports was done separately by the authors. The respective classification was then compared in a four-eye procedure and a consensus was found (Supplementary Table S1).

### 2.2 Survey administration and data collection

Data was collected using an online questionnaire designed for this study by the authors. The online questionnaire was conducted using Unipark an established survey software from Tivian for scientific questions. The questionnaire was distributed by email with a non-personal link. The online questionnaire was available in German and French and contained a total of 55 optional questions with filter questions, which were structured according to the following topics: 1) general information, 2) injuries, 3) menstrual cycle, 4) urinary incontinence, 5) contraception, 6) pregnancy, 7) nutrition, and 8) taboo topics. A total of 1,092 female elite athletes from 107 sports were emailed and invited to participate in the study. Of these, a total of 408 female elite athletes completed the online questionnaire. The questions on contraception were answered by 401 of 408 female elite athletes. The questions on menarche were answered by 388 of 408 female elite athletes with an age statement. The question on secondary amenorrhea was answered by 405 of 408 female elite athletes. All female elite athletes answered the questions about injuries.

### 2.3 Statistical analysis

Frequency data were used to analyse ordinal and nominal scaled variables descriptively. Mean (standard deviation) and median (quartile 1/quartile 3) were determined for metric data. To examine whether subgroups (e.g., lean *versus* non-lean sports, individual *versus* team sports) differed in the endpoints tested, Pearson’s chi-squared test was used. The Mann-Whitney U test was used to test group differences and distributions for metric measurement data. The effects of multiple testing were not considered in the descriptive analysis. Statistical significance was defined as *p*-value <0.05.

## 3 Results

### 3.1 Participants characteristics

Out of the 1,092 female elite athletes contacted, 408 responded yielding a high response rate of 37.4%. For the athlete`s answers to be included in the evaluation the first female-specific topic block (menstrual cycle) had to have been answered. Answers like “I do not know” were considered as unspecified data. Half of the female elite athletes were 23 years old or younger, 30% were 24–29 years old and 20% were 30 or older. Concerning training volume, more than 60% of female elite athletes state, that they train 10–20 h weekly (n = 245), 10.8% less than 10 h weekly (n = 41), and 28.8% more than 20 h weekly (n = 112). In the survey, female elite athletes from individual sports (70.4%, n = 287) were more heavily represented than female elite athletes from team sports (29.6%, n = 121) (Supplementary Table S1). Since female athletes with low body weight or slim appearance have a higher risk of delayed puberty, cycle disorders, FAT, and RED-S (Kapczuk, 2017), we have considered body-emphasising sports separately in this analysis. Supplementary Table S2 shows the distribution of the different types of sport in terms of body emphasis. Most female elite athletes in this survey participated in non-lean sports (56.6%, n = 231), whereby all these 231 female athletes were distributed over 55 sports, which meant certain sports only had a few mentions.

### 3.2 Contraceptive choice among female athletes

Among the Swiss female elite athletes surveyed, 45.1% (n = 181 of 401) of the respondents used hormonal contraceptives and of those 57.5% (n = 104 of 181) used an oral contraceptive pill (OCP). OCP refers to both progestin-only pills (POP) and combined oral contraceptive pills (COC). Among the remaining female athletes with hormonal contraception, 9.4% used a vaginal (n = 17) ring, 32.0% (n = 58) used a levonorgestrel intrauterine device (LNG-IUD) and 1.1% (n = 2) used a progestin-only implant. For the contraceptive pill, no further differentiation was made between the combined contraceptive pill (COC) and progestin-only pill (POP). 54.9% (n = 220 of 401) of the study participants used no or a non-hormonal contraceptive method. In this survey, non-hormonal methods were not broken down further (e.g., copper IUD, barrier contraceptives, sterilisation, natural family planning, coitus interruptus). For further analysis, the 92 sports were divided into lean sports *versus* non-lean sports and the contraceptives were divided into the following three groups: 1) non-hormonal and no contraception, 2) systemically hormonal contraception (oral contraceptive pill, vaginal ring, progestin-only implant) and 3) hormonal intrauterine devices (LNG-IUD). The LNG-IUD was considered separately due to the low serum LNG concentrations (0.1–0.4 ng/mL) and the consequently negligible systemic effects (ESHRE Capri Workshop Group, 2008). Using this grouping, we examined contraceptive trends in relation to lean *versus* non-lean sports. According to this, female athletes in lean sports (25.4%, n = 44) were significantly less (*p* = 0.049) likely to use systemically active hormonal contraceptives (e.g., OCP, progestin-only implant, vaginal ring) than female athletes from non-lean sports (34.6%, n = 79). Female athletes in lean sports were more likely to prefer an LNG-IUD (17.3%, n = 30 in lean sports; 12.3%, n = 28 in non-lean sports), but not statistically significant (*p* = 0.197) or to report the use of non-hormonal or no contraception. The 181 female athletes were asked about their reason for using hormonal contraception. The following multiple-choice answers were possible: 1) contraception, 2) reduction of dysmenorrhea, 3) cycle control, and 4) reduction of bleeding intensity. The most frequent reason given for hormonal contraception was the desire to use a contraceptive (83.4%, n = 151), followed by the possibility of reducing menstrual cramps (36.5%, n = 66), cycle control in competitions (35.9%, n = 65), and reduction of bleeding volume (27.1%, n = 49). Some female athletes mentioned treatment of acne (1.7%, n = 3), treatment of endometriosis (1.7%, n = 3), iron deficiency (1.1%, n = 2), regular bleeding pattern (1.1%, n = 2), low cost (0.6%, n = 1) and stabilisation of body weight (0.6%, n = 1) as further individual reasons for using hormonal contraception. The question of whether the hormonal contraceptive pill influences performance was answered neutrally to positively by most of the female athletes. In terms of numbers, 31.5% (n = 57) felt no difference, 43.6% (n = 79) could not answer the question (“I do not know”), 14.4% (n = 26) thought their performance was more consistent, 1.1% (n = 2) felt it was less consistent, 7.7% (n = 14) felt more powerful, and only 2.8% (n = 5) felt their performance was worse with hormonal contraceptive methods in general.

### 3.3 Primary amenorrhoea in Swiss female elite athletes

Menarche refers to the age at which menstrual bleeding first occurs. On average, this happens between the ages of 12 and 13 (Karapanou and Papadimitriou, 2010). Primary amenorrhoea refers to the absence of menarche up to the age of 15 with the simultaneous presence of sexual characteristics and normal length growth (ACOG, 2015). In the absence of longitudinal growth and secondary sexual characteristics, this is called primary amenorrhoea from the age of 13. 388 female athletes (95.3%) answered the question about menarche with an age statement. Of these, 11.3% (n = 44) reported primary amenorrhoea, and 88.7% (n = 344) reported timely onset of menarche. In female athletes with primary amenorrhoea, menarche tends to occur later in lean sports than in non-lean sports, with 14.0% (n = 24) *versus* 9.2% (n = 20, *p* = 0.149). When comparing individual and team sports (Supplementary Table S2), there was no difference in the onset of menarche (*p* = 0.861). We performed the same calculation based on BMI and divided the athletes into two groups: the normal/overweight group (BMI ≥18.5 kg/m^2^, 97.2%, n = 383) and the underweight group (BMI <18.5 kg/m^2^, 2.8%, n = 11). In this further examination of menarche onset as a function of BMI (underweight *versus* normal/overweight), no statistically significant differences were found (*p* = 0.611) due to the small number of women with primary amenorrhoea in the underweight group (n = 10). To examine the function of BMI on menarche in even greater detail, new BMI subgroups were defined based on the median of BMI (21.7 kg/m^2^), i.e., female athletes with a low BMI (<21.7 kg/m^2^, 49.5%, n = 195) *versus* female athletes with a high BMI (≥21.7 kg/m^2^, 50.5%, n = 199). Examination of the frequency of primary amenorrohea as a function of this BMI classification showed that primary amenorrhoea occurred significantly more often in female athletes with a lower BMI (frequency of primary amenorrhoea: 15.2%, n = 28) than in athletes with a higher BMI (frequency of primary amenorrhoea: 7.4%, n = 14; *p* = 0.021).

### 3.4 Secondary amenorrhoea and oligomenorrhea in Swiss female elite athletes

Oligomenorrhoea is defined as fewer than nine cycles per year or a cycle length of more than 35 days in adults or more than 45 days in adolescents (ACOG, 2015). Overall, 19.1% (n = 77 of 404) of female athletes reported having fewer than nine menstrual cycles per year. Secondary amenorrhoea is defined as an absence of menstruation for more than 3 months (if menstrual cycles were regular before) or for more than 6 months (if menstrual cycles were irregular before) (ACOG, 2015). A total of 405 participants answered the question about a previous episode of secondary amenorrhoea. In total, 32.1% (n = 130) reported secondary amenorrhoea. Namely, 26.7% (n = 108) reported a previous episode of secondary amenorrhea, and a further 5.4% (n = 22) reported a current episode of secondary amenorrhoea. This figure was consistent with the answers to the question about the last menstruation. Thus, 2% (n = 8) of the female athletes stated that their last menstruation was 3–4 months ago and another 2.5% (n = 10) stated that their last menstruation was more than 5 months ago.

### 3.5 Injuries in Swiss female elite athletes

All female athletes surveyed answered the question about an injury in the past year. 57.1% (n = 233) of the respondents reported that they had not been injured, while 32.6% (n = 133) reported at least one injury and 10.2% (n = 42) reported two or more injuries per year. The risk of injury did not differ when comparing lean *versus* non-lean sports. We also examined the influence of training volume and secondary amenorrhoea on the frequency of injuries. A total of 398 athletes reported their training volume, and 387 athletes reported both their training volume and information about a current or past episode of secondary amenorrhoea.

To investigate differences in the incidence of injuries in female athletes with secondary amenorrhoea, we divided the data of these 387 female athletes into two groups: 1) less than 21 h of training per week (71.9%, n = 279) and 2) at least 21 h of training per week (28.1%, n = 108). Female athletes affected by secondary amenorrhoea and with high training volumes (≥21 h/week) were significantly more likely to report at least one injury per year (n = 29 of 41; 70.7%), whereas female athletes with a current or past episode of secondary amenorrhoea and lower training volumes (<21 h/week) tended to report fewer injuries (n = 37 of 87; 42.5%, *p* = 0.004). In contrast, there were no statistically significant differences in women without secondary amenorrhoea regarding injuries at different training intensities (39.6% vs 38.8%, *p* = 1.000). Accordingly, female athletes in lean sports with a current or past episode of secondary amenorrhoea and higher training volumes (≥21 h per week) had a significantly higher risk of injury.

## 4 Discussion

Our online cross-sectional survey of 92 different sports in Swiss female elite athletes shows that the incidence of primary amenorrhoea (11%), secondary amenorrhoea (5.4%), and oligomenorrhoea (19.1%) in Swiss female elite athletes remains a problem compared to the incidence in the normal population (Gasner and Rehman, 2022; Lord and Sahni, 2022; Riaz and Parekh, 2022) and also compared to the last survey in 2001 (Matter and Marti, 2001). Compared to the general population in Switzerland, considerable proportion of the female elite athletes surveyed used non-hormonal contraception or no contraception at all. Injuries were particularly common especially in female athletes affected by secondary amenorrhoea and with higher training volumes. All these observations in female athletes (cycle irregularities, contraceptive choice, and injuries) were related to the nature of the sport (lean *versus* non-lean). With 408 elite female athletes participating, the results reflect a representative sample of Swiss elite athletes. In the context of the informal request for study participation within a newsletter by email through Swiss Olympic, the response rate of 37% is be regarded as significant.

### 4.1 Female cycle disorders

The question about the first menstrual bleeding was answered with an age statement by 95% (n = 388 of 408) of Swiss female elite athletes. Overall, 11.3% of the Swiss female elite athletes surveyed stated, that their menstruation started late, i.e., after the age of 14, which corresponds to primary amenorrhoea (ACOG, 2015). According to the latter survey of Swiss female elite athletes in 2001, 0.3% (3/157) of the female elite athletes surveyed reported primary amenorrhoea. Trends in the onset of menarche showed that it tended to occur later in female athletes competing in lean sports (14.0%) compared to non-lean sports (9.2%). A recent survey of 1,020 female collegiate athletes in the USA provided similar figures, with 11.9% (n = 123) of female athletes having primary amenorrhoea (Cheng et al., 2021). Similar findings were also seen in other studies (Beals and Hill, 2006; Sundgot-Borgen and Larsen, 2007), as well as in a cross-sectional study of female high school athletes (n = 423) in which the prevalence of menstrual irregularities was significantly (*p* = 0.010) higher in lean sports (26.7%) than in non-lean sports (16.6%) (Nichols et al., 2007). Our results show that female athletes in lean sports with a high training volume (≥21 h/week) were affected by primary amenorrhoea (19.0%) significantly more frequently than female athletes from non-lean sports (4.1%) with the same training volumes. The influence of training volume on menarche was described in a prospective observational study, in which a correlation between reduction in training volume and progression of sexual development and the onset of menarche was observed in 10 out of 15 female ballet dancers during a 2-month training break (Warren, 1980). Athletes who have high training volumes or compete in lean sports (Torstveit and Sundgot-Borgen, 2005b; Schaal et al., 2021) are at greater risk of energy deficiency, which in turn can have a modulating effect on the HPG axis, prolonging the prepubertal state and causing primary amenorrhoea. The maturation of the gonadal axis depends on the age at which menarche occurs. Between the ages of 12 and 13, it takes about 3 years for the cycle to become established with a stable cycle length and regular ovulatory cycles (>50%) (Apter and Vihko, 1983). Delayed menarche, therefore, entails a significant delay in the maturation of the gonadal axis, so it takes a further 8–12 years on average for the cycle to stabilise. Besides delayed maturation of the HPO axis, exercise-related delayed menarche may have consequences for growth velocity, peak bone mass acquisition, and bone microarchitecture (Rudolph et al., 2021). 396 participants answered the question about a previous episode of secondary amenorrhoea. In total, 32.8% reported secondary amenorrhoea, i.e., 26.5% reported a previous episode of secondary amenorrhoea, and 5.4% reported a current episode of secondary amenorrhoea. In answer to another question, 19.1% reported an oligomenorrhoea, of which 67.5% where within the context of secondary amenorrhoea. The proportion of female athletes with secondary amenorrhoea in our survey was probably high but is comparable to results from other cross-sectional surveys of other countries, with prevalence ranging from 1.4%–69% in lean sports to 0%–12.8% in non-lean sports (Javed et al., 2013). A survey of female elite athletes in Denmark revealed the highest prevalence of 69% for secondary amenorrhoea in endurance athletes, where amenorrhoea was associated with higher cardiovascular training volumes (Oxfeldt et al., 2020). This wide range of prevalence varies depending on the type of sport, different classification criteria, training volume, assessment of contraception, and the different age groups of the female athletes surveyed (Nattiv et al., 2007). In comparison, the prevalence of amenorrhoea in the normal population is 3%–4% (Bachmann and Kemmann, 1982). Prolonged primary or secondary amenorrhoea is a serious condition that should be addressed medically as it can affect multiple body systems (including bone microarchitecture) and is associated with a greater likelihood of stress fractures in female athletes (Ackerman et al., 2011). In addition to the more frequent occurrence of amenorrhoea and the associated risk to bone health, female athletes in lean sports tend to seek medical help less frequently on their own (Zawila et al., 2003; Miller et al., 2012). Regular medical follow-up with screening for RED-S and FAT symptoms is therefore essential for female athletes engaged in lean sports.

### 4.2 Contraception

The analysis of our results showed that most female athletes either did not use contraception at all or used non-hormonal contraception (54.9%). An OCP was used by 25.9% of the female athletes (COC or POP), 14.5% used an LNG-IUD, and 4.2% a vaginal ring. Compared with the normal population in Switzerland, it seems that OCPs are less popular among Swiss female elite athletes. According to the 5-yearly representative survey of 3,555 Swiss women (aged 15–49) in 2017, the condom was used most frequently (42%), followed by the OCP (31%; POP + COC) (Späth CS et al., 2017). This trend of less frequent use of oral contraception among Swiss female elite athletes in this survey was also observed in a recent cross-sectional survey of female elite Australian athletes, which found that 26.5% of female athletes (Clarke et al., 2021) *versus* 43.2% in the general population (Skiba et al., 2019) used OCPs. This less frequent use of OCPs among female athletes compared to the normal population has not been found in other countries, such as Denmark, where 57% of the athletes reported using OCPs (41.3% COC, 3.7% POP) (Oxfeldt et al., 2020), compared to 42% within the general population (Lindh et al., 2017). Similar results have been reported by surveys among elite athletes in the United Kingdom (Martin et al., 2018), Norway (Torstveit and Sundgot-Borgen, 2005a)^,^ and the USA (Verrilli et al., 2017). In the United Kingdom, for example, female athletes are more likely to use OCPs (34.5%) compared to the general population (30%) (Cea-Soriano et al., 2014; Martin et al., 2018). The contraceptive situation in Norway, Portugal, and the USA is similar; 40.2% of female athletes in Norway (Torstveit and Sundgot-Borgen, 2005a), 72% of female athletes in Portugal (Coutinho et al., 2021)^,^ and 57% of female athletes in the USA use OCPs (Verrilli et al., 2017; Cheng et al., 2021), compared to 27% in the Norwegian population, 30.9% in the Portuguese (United Nations DoEaSA and Population Division, 2019) and 14% in the US (Lindh et al., 2017). These different tendencies suggest that attitudes towards contraceptive methods may vary between countries due to sociocultural, geographical, individual or other influencing factors (Clarke et al., 2021). Combined hormonal contraception reduces free androgen serum concentrations (e.g., free testosterone) through induction of hepatic proteins, namely, sex hormone binding globuline (SHBG) (van der Vange et al., 1990; Zimmerman et al., 2014). Because of this, the question is often raised whether COCs could have a performance-reducing effect (Crewther et al., 2018). The influence of hormonal contraception on objectifiable athletic performance was examined in a recent systematic review with the meta-analysis by Elliott-Sale et al. (Elliott-Sale et al., 2020). In this article, a subtle reduction in athletic performance was objectively found with OCPs. The relevance of these results for daily life must be questioned, according to Elliott-Sale et al. It should be added, however, that the included studies (n = 42) were very heterogeneous and only 17% were classified as “high-quality”.

### 4.3 Injuries

All female athletes surveyed answered the question about an injury in the past year. 57.1% of the female athletes reported that they had not been injured, 32.6% reported at least one injury and 10.2% reported two or more injuries per year. Female athletes in lean sports with a current or past episode of secondary amenorrhoea who trained intensively (≥21 h per week) had a significantly higher reported incidence of injury. Other studies have also shown that female athletes in lean sports are at greater risk of injury (Nattiv et al., 2007; Mountjoy et al., 2018). According to the literature, female athletes are more often affected by overuse injuries than men (Frank et al., 2017), whereas male athletes are more often afflicted by traumatic injuries (Stracciolini et al., 2014). A chronic energy deficiency can influence the risk of injury, as it interacts with hormones of the somatotropic regulatory circuit (e.g., anabolic effects through insulin-like growth factor IGF-1) and influences musculoskeletal tissue turnover (e.g., regeneration potential of connective tissue, protein synthesis) (Frank et al., 2017).

## 5 Conclusion and perspectives

The results of this orienting survey among Swiss female elite athletes show that contraceptive behavior in Swiss female elite athletes differs in comparison to the normal population. The majority of Swiss female elite athletes used no or non-hormonal contraceptive methods.

The reason for different contraceptive choices is yet to be determined. According to the literature, a possible explanation for a declining rate of hormonal contraceptives could be a lack of information in both the women and the care-taking physicians concerning the needs of each individual patient (Claringbold et al., 2019). Furthermore, a large proportion of the female athletes interviewed reported having had or still having contact with cycle disorders. The incidence of cycle disorders (primary amenorrhoea, secondary amenorrhoea and oligomenorrhea) has increased compared to the normal population and is even higher in percentage of elite athletes compared to the last survey in 2001. Due to the later risk of reduced bone density in female athletes with past primary or secondary amenorrhoea in their teenage years, gynecological-endocrinological assessment, longitudinal observation, and treatment if needed are essential. The main results of this orienting survey of Swiss female elite athletes underline the importance of specialized gynecological care for female high-performance athletes.

## 6 Limitations

The proportion of underweight female athletes (BMI <18.5 kg/m^2^) appears to be very low in this survey (2.8%, n = 11). If the proportion of underweight female athletes is underrepresented due to missing data, an even higher proportion of female athletes with primary or secondary amenorrhoea would be expected. When choosing a different BMI classification using the median (BMI <21.7 kg/m^2^ *versus* BMI >21.7 kg/m^2^), a statistically significant accumulation of delayed menarche depending on BMI could be demonstrated (*p* = 0.021). This finding would have to be substantiated in larger samples. Another limitation of this study is the lack of assessment of energy deficiency among the Swiss female elite athletes surveyed. In the questions on OCPs, no further distinction was made between combined (COC) and progestin-only pills (POP) or other contraceptive methods such as, for example, the hormonal patch, for example,. Similarly, the non-hormonal contraceptive methods lacked a differentiation between the various non-hormonal methods (e.g., copper IUD, diaphragm, condom, natural family planning, coitus interruptus). According to the literature, lean sports are associated with an increased risk of RED-S-related symptoms such as cycle disorders and injuries (Kapczuk, 2017). The aim of the classification of sports into lean *versus* non-lean sports served to group the study participants with an increased risk for cycle disorders and injuries. Since some sports fulfill aspects of both lean and non-lean sports (e.g., pentathlon, shot put in athletics), a certain residual blurring of the distinction could not be completely avoided.

## Data Availability

The raw data supporting the conclusion of this article will be made available by the authors, without undue reservation.
