# Supplementary material for: Contraception, female cycle disorders and injuries in Swiss female elite athletes—a cross sectional study
Source: Front Physiol. 2023 Jul 26;14:1232656. doi: 10.3389/fphys.2023.1232656 (PMC10410265; doi:10.3389/fphys.2023.1232656)
Supplement: Supplementary file 2 [file Table2.DOCX]

**Supplementary Table 2.** Classification of sports into “lean sports” and “non-lean sports”

| **Non-lean sports** | **Count (n)** | **Frequency (%)** |
| --- | --- | --- |
| Badminton | 2 | 0.5 |
| Archery | 2 | 0.5 |
| Base-/softball | 4 | 1 |
| Basket 3x3 | 3 | 0.7 |
| Basketball | 4 | 1 |
| Beach volleyball | 4 | 1 |
| Beachsoccer | 6 | 1.5 |
| Billard | 1 | 0.2 |
| Cycling (BMX) | 1 | 0.2 |
| Bob | 3 | 0.7 |
| Canoe polo, regatta, slalom | 7 | 1.7 |
| Chess | 2 | 0.5 |
| Curling | 9 | 2.2 |
| Equestrian dressage | 3 | 0.7 |
| Equestrian eventing | 5 | 1.2 |
| Fencing | 8 | 2 |
| Field hockey | 7 | 1.7 |
| Fistball | 5 | 1.2 |
| Floorball | 10 | 2.5 |
| Football | 9 | 2.2 |
| Golf | 3 | 0.7 |
| Handball | 4 | 1 |
| Ice hockey | 7 | 1.7 |
| Ice stock sport | 3 | 0.7 |
| Inline speedskating | 1 | 0.2 |
| Kendo | 3 | 0.7 |
| Kick boxing | 2 | 0.5 |
| Lifesaving | 9 | 2.2 |
| Luge | 1 | 0.2 |
| Miniature golf | 2 | 0.5 |
| Motorcycle sport | 1 | 0.2 |
| Parasports | 3 | 0.7 |
| Pentathlon | 2 | 0.5 |
| Rugby | 5 | 1.2 |
| Sailing | 1 | 0.2 |
| Shooting sports | 12 | 2.9 |
| Skeleton | 1 | 0.2 |
| Ski (alpin) | 20 | 4.9 |
| Skydive | 1 | 0.2 |
| Snowboard | 7 | 1.7 |
| Squash | 2 | 0.5 |
| Table tennis | 2 | 0.5 |
| Ski (telemark) | 2 | 0.5 |
| Tennis | 11 | 2.7 |
| Cycling (track) | 2 | 0.5 |
| Cycling (TRIAL) | 1 | 0.2 |
| Tug | 4 | 1 |
| Ultimate | 8 | 2 |
| Volleyball | 4 | 1 |
| Wake board, water skiing | 4 | 1 |
| Water polo | 6 | 1.5 |
| Wildwater canoeing | 1 | 0.2 |
| Wrestling | 1 | 0.2 |
| **Total** | **231** | **56.6** |

| **Lean sports** | **Count (n)** | **Frequency (%)** |
| --- | --- | --- |
| Acrobatics | 1 | 0.2 |
| Cycling (artistic) | 2 | 0.5 |
| Artistic roller skating | 2 | 0.5 |
| Swimming (artistic) | 6 | 1.5 |
| Athletics | 31 | 7.6 |
| Biathlon | 8 | 2 |
| Cycling (orienteering) | 1 | 0.2 |
| Ski (cross-country) | 7 | 1.7 |
| Dancing | 4 | 2 |
| Diving sport | 2 | 0.5 |
| Duathlon | 1 | 0.2 |
| Endurance riding | 6 | 1.5 |
| Equestrian show jumping | 1 | 0.2 |
| Equestrian vaulting | 4 | 1 |
| Figure skating | 2 | 0.5 |
| Gymnastics (artistic) | 11 | 2.7 |
| Judo | 3 | 0.7 |
| Ju-jitsu | 3 | 0.7 |
| Karate | 7 | 1.7 |
| Mountain running | 3 | 0.7 |
| Cycling (mountain bike) | 10 | 2.5 |
| Orienteering | 8 | 2 |
| Paragliding | 1 | 0.2 |
| Gymnastics (rhythmic) | 1 | 0.2 |
| Cycling (road) | 3 | 0.7 |
| Rowing | 14 | 3.4 |
| Ski (freestyle) | 4 | 1 |
| Ski (orienteering) | 1 | 0.2 |
| Ski (touring) | 2 | 0.5 |
| Speed skating | 1 | 0.2 |
| Sport climbing | 5 | 1.2 |
| Swimming | 5 | 1.2 |
| Synchronized skating | 5 | 1.2 |
| Taekwondo | 3 | 0.7 |
| Triathlon | 8 | 2 |
| Twirling | 1 | 0.2 |
| **Total** | **177** | **43.4** |
